# “We’re living in a world that wasn’t built for us”: A qualitative exploration of young New Zealander’s perspectives on socio-ecological determinants of declining youth mental health

**DOI:** 10.1186/s12889-025-22618-2

**Published:** 2025-05-05

**Authors:** Jessica Stubbing, Kerry Gibson, Anne Bardsley, Peter Gluckman

**Affiliations:** 1 Koi Tū: The Centre for Informed Futures, The University of Auckland, Auckland, New Zealand; 2 School of Psychology, The University of Auckland, Auckland, New Zealand

**Keywords:** Youth, Mental health, Prevention, Early intervention, Social determinants

## Abstract

**Background:**

Globally, youth mental health has been in decline since the beginning of the 21st century. While much has been written about the reasons for this, we have little understanding of young people’s perspectives. A rich understanding of the issues young people identify as impacting their mental health is essential for developing effective interventions. This study aimed to explore what young people from Aotearoa, New Zealand believe impacts their mental health, both positively and negatively, with a focus on social determinants.

**Methods:**

176 young people (16–25-year-olds, mean age 17) participated in one of 19 workshops held in 2023. Our methodology was informed by participatory research methods and developed in consultation with young people who served as advisors. Workshops were held across rural and urban areas of two regions of New Zealand. Recordings were transcribed, combined with survey responses, and analysed through Reflexive Thematic Analysis.

**Results:**

Four themes were identified which young people perceived as impacting their wellbeing: *The world we live in*,* the pressures we experience*,* the connections we need*, and *finding our path.* All factors identified as impacting on their mental health, both positive and negative, were ‘amplified’ by social media.

**Conclusions:**

Our findings highlight the intersecting issues of systemic social determinants of wellbeing and their complex relationship with an evolving digital landscape. Effectively addressing rising rates of mental health challenges is likely to hinge on both population level action to address social determinants and targeted promotion of strategies to support young people to navigate the increasing complexity of modern life.

**Supplementary Information:**

The online version contains supplementary material available at 10.1186/s12889-025-22618-2.

## Background

Around the world, research consistently indicates high rates of impaired mental health and well-being among young people [1, 2]. Perhaps even more concerningly, a recent decline in youth mental health and well-being has been documented across countries where data is available (e.g., [3, 4–8]) and was documented before the COVID-19 pandemic, which has negatively impacted youth wellbeing (e.g., [9, 10]).

Beyond the moral imperative to address these trends to reduce suffering for young people, improving youth mental health is also a public health priority. Mental health during youth has important implications for lifelong outcomes in mental health, social functioning, education, and the workforce among others [11–15]. Mental health also has significant impacts on economic outcomes, productivity, and spend in the healthcare sector [16]. Sustainably improving mental health and in turn these lifelong outcomes for young people is essential. Though intervention and treatment for young people experiencing major mental health challenges are important, no mental health system is successfully and sustainably responding to the rapidly increasing population in need of support. This highlights the need to improve our focus on prevention and early intervention: improving our understanding of *why* mental health is declining for young people, addressing modifiable risk factors at the source, and building on existing opportunities for positive change.

Researchers have an increasingly strong grasp on what influences mental health including biological, psychological, and developmental determinants. Most life course mental health challenges have onset before 25, highlighting the importance of youth in the development of mental health challenges [17–19]. Biological systems important for the development of mental health challenges include the HPA-axis, involved in regulating stress response, and the prefrontal cortex, associated with executive functioning [17]. The neurodevelopment of such systems is complex and significantly influenced by the social environment, particularly during sensitive periods throughout prenatal, infancy, childhood, and adolescent periods [20] with many hypotheses as to how these interactions and intersections play out neurologically [19, 21]. Social environmental influences that can impact development negatively include chronic stress, low socioeconomic status, neglect, parenting approaches, lack of adequate nutrition, exposure to violence, and experiences of abuse [18, 19]. By contrast, social experiences like supportive relationships can also positively impact neurodevelopment [18].

While we may understand some of these factors that influence the development of mental health challenges in youth, we do not have a thorough grasp of current and evolving social determinants and the role these might play in undermining young people’s wellbeing. In the attempt to account for the rapid decline in youth mental health in recent decades, researchers have pointed to rapidly changing social conditions [22]. Sociologists have documented changes across a range of variables documented to affect mental health and wellbeing including housing [23], socio-economic status [24, 25], experiences in education [26, 27], access to healthcare [28], political decision making [29, 30], and support for families [31]. The rise in social media in particular has drawn significant attention for the influence it may have on youth mental health (e.g., [32]). Others have drawn attention to the social and economic pressure young people face in neoliberal societies, like Aotearoa New Zealand, and the negative impact this has on mental health [33]. More recently researchers have recognised the impacts of existential threats such as pandemics like COVID-19 [34] and climate change [35].

The rising rates of mental health problems amongst young people have been a significant concern in Aotearoa New Zealand (NZ; 36). The Aotearoa/NZ social and political context has much in common with other OECD countries but also has one of the most diverse populations of young people, which includes the indigenous Māori, the descendants of European settlers, indigenous people from the broader Pacific islands surrounding NZ (Pasifika), and a range of migrants from other countries. Research in Aotearoa/NZ has noted disproportionate rates of mental health problems amongst Māori, Pasifika and other minoritised ethnic groups, linking this to persistent socio-economic deprivation over generations, as well as the structural and interpersonal racism faced by these groups of young people [37, 38].

It is well recognised that social determinants that negatively affect mental health are unequally distributed across countries and populations. These are not accidental differences but are a product of strategic decision-making which influences access to power and resources necessary for wellbeing [39]. While Aotearoa/NZ forms part of relatively privileged group of OECD countries and might be assumed to share their advantages, this position hides significant pockets of disadvantage that might be comparable to poorer countries. Aotearoa/NZ has relatively high rate of poverty compared with other countries and Māori and Pasifika children experience poverty at much higher levels (20 and 24%, respectively) than children of European descent (8%) [40].

While there is a growing body of research that suggests that young people are facing increasingly challenging social contexts, there is a need to understand which particular aspects are salient to young people and why these matter to them. In order to address this gap in understanding, it is important to develop our knowledge of young people’s own experiences of these social determinants and their perspectives on how these influence their well-being.

Typically, research on the contribution of social determinants to mental health challenges will link broad scale changes in society to mental health prevalence in particular groups [41]. It is less common to examine social threats to mental health from the perspective of those who are subject to these. This is particularly the case for young people, who have often been excluded from research that concerns them [42]. There are, however, important reasons for considering young people’s own views on the social conditions that might be affecting their mental health. It is difficult to establish the significance of social changes for young people without considering the ways in which these are understood from within the youth cultures that define their significance. This is particularly important as youth cultures are changing rapidly, and it is crucial that research keeps up with the changing pressures that young people perceive in their social worlds. There is also increasing awareness that adults, in general, are not always privy to the issues that the younger generation are dealing with, and young people have expertise in understanding youth cultures and experiences that adult researchers often lack. Finally, the shift to recognising the expertise of young people on issues that concern them coincides with a growing political awareness of the need to listen and respond to young people’s voices in society more generally. While there has been some interest in examining some of challenges that young people experienced during the COVID-19 pandemic [43], there is a dearth of ecological research that captures the social factors young people see as being detrimental to their mental health more generally. It is vital that researchers enlist the expertise of young people to help us identify and address aspects of their social environment that might be impacting negatively on their mental health.

### Objective

The present study aims to investigate young people’s views on the social determinants of rising rates of mental health distress in Aotearoa/NZ, including what they see as helping and harming. Using a large-scale participation research approach across diverse population groups of young people, we aimed to explore what young people see as the most important social issues and areas of need for them.

## Method

### Study Design

This research was grounded in a critical empowerment perspective of youth, focused on centring the lived experiences and expertise of young people. Data was collected using a participatory workshop method, designed in previous research [44] which was adapted for the current study collaboratively with a Rangatahi (youth) Advisory Committee. This workshop method provided multiple ways for young people to express their views on the social conditions they perceived to impact negatively and positively on their mental health, and to shape the researchers’ understanding of their priorities and concerns. This approach was developed to ensure that youth voice is adequately represented and to address the power-imbalances that often silence young people on issues that concern them.

### Advisory group

Members of the Rangatahi advisory group were invited through the broader departmental Rangatahi advisory group. One chair of this advisory group was closely involved in recruiting members of this group and consulting with the research team on the group’s role and scope. Members were required to identify as rangatahi and to be in the Auckland region for meetings, there were no other criteria for membership. All members were paid for their contributions to the project. Members of the group represented a wide range of young people of various ages, genders, ethnicities, and backgrounds. The Rangatahi advisory group contributed to the project through defining research questions and scope of the project, designing the workshop and all data collection activities within this, participating in a mock workshop to further develop and refine the workshop process and all activities, contributing to survey design and wording, consultation and design of recruitment strategies including outreach procedures, decisions regarding when to conclude data collection, and the interpretation and presentation of findings.

### Participants

Theoretical sampling was used to identify young people aged 16 to 25 years who represented the diversity of Aotearoa/NZ society, including Māori, Pacific Islanders, migrants, and the disability and LGBTQ+ communities. To facilitate a mix of urban and rural perspectives, recruitment was conducted in the Auckland and Northland regions of Aotearoa/NZ, including both urban and rural areas. As part of a commitment to community involvement and to facilitate recruitment, the researchers developed partnerships with a range of organisations (iwi groups, community groups, and clubs) in these regions who assisted with recruitment. Recruitment was done via direct communication from the organisations to their members or through their social media networks. Partner organisations hosted the workshops, usually with support of an adult champion. In developing our workshop procedure and through consultation with the Rangatahi advisory committee and community partners, we identified that it was beneficial for young people to be able to attend with other members of their community including those they know well. This fostered whanaungatanga, a term in Te Ao Māori that refers to a sense of belonging and connectedness, that could support feelings of safety and promote engagement. To ensure participants could share perspectives they may not have felt able or willing to share with others they know present, participants were given opportunities to share feedback privately and anonymously through surveys.

176 young people aged 16–25 (*M* = 17.61, *SD* = 2.28) participated in these workshops which were held between February and September 2023. Workshop sizes varied significantly, from 4 to 18 participants (*M = 9.3*,* SD = 4.2)* Table 1 describes participant demographics.

**Table 1:** Participant demographics and description

	Number
Ethnicity*	
New Zealand European/ Pakeha	69
Māori (indigenous people of New Zealand)	53
Pacific Islander	48
Asian	23
MELAA (Middle Eastern/Latin American/African)	7
Other	3
*Nb. Many participants identified with multiple ethnicities. As such,* these numbers do not add to 176.*	
**Gender**	
Male	71
Female	96
Gender Diverse/Gender Queer/Non-binary	9
**LGBTQIA + Identity**	37
**Lived experience with mental health challenges**	135
**Lived experience of supporting others with mental health challenges**	143
**School and work**	
Engaged in school or university	146
Engaged in work	85
Neither engaged in education or work	6

### Ethics

Ethics approval was granted by the Auckland Health Research Ethics Committee. All participants provided written informed consent. This research adhered to the Helsinki Declaration. Given the sensitive nature of this research, considerable care was taken to ensure psychological safety for participants. This included monitoring conversations for acute distress and establishing procedures to support young people in need of intervention. Participants were provided with contact information for support services should they need them, and a psychologist or trained clinician was present or on site to provide support or guidance as necessary. Each session ended with a transition activity, debriefing and reflecting as a group on the content of the workshop and easing into discussion of ‘life outside of the workshop’; and a karakia, a traditional way of calling a close to proceedings in Te Ao Māori. Following each session, the research team remained on site and encouraged participants to approach them for debriefing, support as needed, or for more casual conversation. These procedures were designed to call a formal close to the workshop proceedings, support participants to transition safely from the content discussed and ensure opportunities to identify and respond to any distress expressed. Those who requested so were provided with contact information for services and/or connected with an identified support person within the organization who supported recruitment for that workshop if the participant desired this.

### Data Collection

Data was collected and partially analysed through the participatory workshops that drew on young people’s own expertise of mental health. The workshop was designed to enable an iterative process through which key issues affecting mental health could be identified, discussed and refined through various activities and discussion. The workshops began with individual surveys to promote private reflection and space to share responses that might be considered socially undesirable. Surveys, developed with our advisory group, are included in Appendix 1. This was followed by a ‘focus group’ style discussion. Following this, participants completed small group activities including creating mind maps and digital maps, during which they responded to questions aimed at clarifying the key issues that had emerged during the earlier discussions and providing a further opportunity to develop these ideas. The workshop concluded with participants completing individual questionnaires to reflect their current understanding of the main factors contributing to youth mental health problems.

The diverse activities incorporated in these workshops were intended to be engaging and interactive, promoting participation from as wide a range of young people as possible, allowing for multiple approaches to expression. While participants were invited to discuss issues in relation to young people in general, they often referred specifically to their own experiences with mental health.

Dependent on group size, workshops lasted between two and three hours and were facilitated by research assistants and the lead author. Both the large and small group discussions were audio-recorded and fully transcribed, and the digital maps were stored electronically for reference.

### Analysis

The analysis followed a two-phase process. In the first phase the researchers used the data generated from the digital maps to identify priority issues affecting youth mental health as identified by the workshop participants. These digital maps were constructed with Ako maps [45], educational software previously utilized in research [46], which was used to construct live maps during the workshops which participants could directly contribute to, provide feedback on, and edit live. The researchers collated this list and identified those that were most commonly reported during the workshops. Content related to discussion on these was extracted from the transcripts for analysis. In the second phase, data was analysed following Braun and Clarke’s framework for reflexive thematic analysis [47]. This process involves a reflexive process of theme development to capture the issues raised by participants and the way in which they understood their significance.

The priority issues identified by participants showed some overlap and repetition, providing a starting point for the analysis. For example, the issue of social media was clearly recognised as significant for mental health and was often discussed as an amplifier in relation to both positive and negative influences on mental health. The impact of COVID-19 also cut across several of the themes as did issues related to diversity and inequality in NZ society. To account for this, we worked iteratively between the list of factors identified in the digital maps and transcribed discussions to synthesise themes that reflected the key ideas raised by the participants.

Due to the extremely large data set produced, data analysis began with immersion in a subset of the data by authors 1 and 2 to generate initial codes and preliminary ideas. Immersion in the full data set followed, with all transcripts read several times. All statements related to influences on youth mental health were extracted, and tentatively grouped into overarching categories and assigned codes. These were then shaped into themes. This process was not linear and was iterative, with contributions from all authors and multiple research assistants until it was felt to accurately reflect the data. NVIVO 12 software was utilised for support. Examples illustrating each theme were then extracted from the transcripts. Descriptors such as ‘most’, ‘some’, and ‘many’ are included to indicate how often concepts within themes were discussed. These are not intended to imply statistical measurement.

Our process of discussion, review, and refinement throughout the analysis was important for increasing fidelity to the stories we heard, improving the rigour of our analysis, and ensuring our final themes were a fair and accurate reflection of the data not overshadowed by author perspectives [48]. Reflexivity was an important practice throughout our analysis, involving all authors acknowledging the contextual nature of meaning making and remaining attentive to our own perspectives and how these influence our interpretation of data. In particular, we remained aware of our professional positioning (authors 1 and 2 are psychologists with experience working clinically with young people) and personal perceptions of young people (most research assistants on the study are young people within the age range of participants) and prioritised questioning our assumptions and resisting urges to ascribe young people’s stories to mainstream discourses of mental health and its determinants. Additionally, we kept digital records throughout our analytic process to promote transparency and communication.

### Findings

**Fig. 1:**
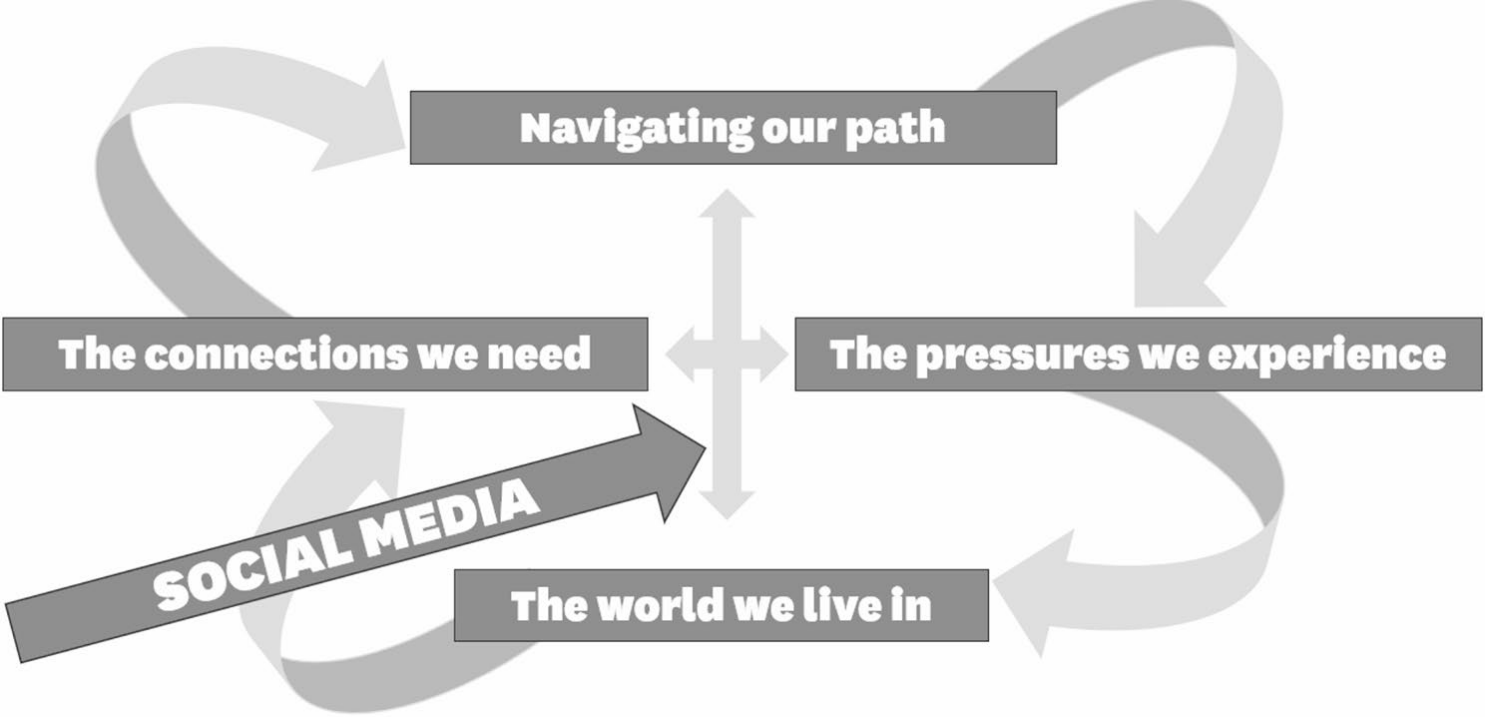
Graphic illustration of interconnections between the four themes and social media.

Four main themes were identified through our thematic analysis which broadly synthesised the multiple influences participants identified as impacting young people’s mental health. Importantly, though these themes are presented separately for clarity, all themes had significant overlap and no participants believed one single factor was responsible for mental health. Participants’ understanding was sophisticated and nuanced, showing a systemic appreciation of determinants of mental health. Figure 1 illustrates the interconnections between the four themes, and the role social media plays contributing to each of these.

#### 1. The world we live in: “The world is deteriorating”

Across all workshops, participants discussed the state of the world and how this impacted mental health and wellbeing for young people. Participants sensed a world fraught with political, social, and economic problems, and articulated concerns about what this meant for their own futures. Core concerns included uncertainty about their economic futures, existential threats, and social problems exacerbated by polarisation, inter-generational divides, and inequality. Conversations were often characterised by a feeling of being overwhelmed and fatigued by the state of the world:

Even since from our childhood, so many once in a lifetime occasions. Financial crisis, global pandemic…Housing crisis, youth mental health crisis.

Participants expressed frustration with the economy, often centred around the cost of living and housing security. There was considerable discussion about the economic difficulties confronting their generation and inequalities which compounded the problem. Some young people were clearly facing considerable economic pressures in their current situations. One young person described how they battled to cover their own living expenses each week:

My wage. It’s like it’s like, I feel hopeless. Yeah. And I feel exhausted. I feel so tired… I only slept like four hours.

Other young people conveyed an awareness of their privilege relative to others but felt uncomfortable with the clear inequalities they saw around them. There was, for example, discussion about how colonisation had disadvantaged Māori and recognition that other marginalised groups were also unfairly disadvantaged. There was, however, a pervasive sense that regardless of privilege no one was immune from uncertain economic future:

No one knows if they’re gonna get a job after school or how the job is gonna go.

Discussion in the workshops often reflected anger against those who seemed oblivious to these social and economic problems. They spoke about their frustration with an ideology that promoted wealth and perpetuated a false sense of how people could and should live:

I know it’s called the American dream, but it’s sort of applicable anywhere that has capitalism. That you need to chase money, and you need to chase wealth, or you can’t be happy and can’t enjoy the simple things in life.

It was common to hear both pessimism and cynicism in discussions about the economy:

I’m concerned about just the spiralling economy and you know, heading into potentially a period of bad …. there’s alreadtime that could be really y a lot of poverty. Like, you know, maybe the working-class people won’t be able to buy food anymore. Like it might be, like the Great Depression.

The economy was not the only source of uncertainty, with participants also describing stress related to existential threats like climate change and global wars:

Things we … hear about, like the war in Ukraine and the climate crisis…. Those two aspects have an impact on mental health. They contribute to a sense of pessimism among the younger generations that the world is deteriorating.

Within this context, many participants described difficulty picturing their future, often described significant anxiety and concern:

Like having kids is stressful and like scary to think about. You second guess like the idea of it.

These conversations did not solely centre on the future: young people were also deeply concerned about how the state of the world impacts mental health in the present. Issues discussed in this context included substance use and exposure to violence. There was a particular discussion on how living conditions impact mental health, with many feeling good mental health is contingent on basic living conditions:

They don’t have somewhere to live, riddled with mould, it’s overcrowded, they can’t access the doctor…they aren’t able to feed themselves. They don’t have anyone to talk to. So, obviously, you’re more likely to have mental health issues.

Others resisted this narrative, noting that young people still struggle with their mental health when these basic needs are met:

They seem to think, well, if we shell out more money for the minimum wage and stuff like that our kids will get better because they’re going to have shoes on their feet, lunch in their lunchbox. But that’s not how it works.

Discrimination including sexism and racism were also discussed. Many young men described feeling left behind in society, and that there is not enough attention to men’s mental health and wellbeing. Many young women and femme presenting youth discussed how experiences of sexism and gender-based violence impacted mental health, with some describing catcalling and harassment:

Half the things men say to me, half the things they do when they walk past me on the street …. That impacts my mental health because it makes me feel unsafe.

Inter-generational division was also commonly discussed, with many participants expressing frustration with how previous generations have led to them ‘inheriting’ a broken world. While some of the participants recognised that their parents had also suffered, there was a tendency to direct anger towards the older generations for being oblivious or unresponsive to the struggles that young people were facing:

What is wrong with these old people? Like, do they really not educate themselves about anything that’s going on in this world right now?

Notably, participants in the workshops described a feeling of helplessness to address the problems they saw in their society, and frustration that adults did not take their perspective seriously:

They think because we’re younger, we’re not wise enough. It’s like, ‘Oh, you’re just young, you don’t know anything.’ I think that’s pretty dumb.

Other forms of division were also commonly discussed, including awareness of rising polarisation in society:

The media has never been more divided than it is at the moment. You’re on one side, or you’re on the other. I’ve very rarely come across anyone who’s moving towards the middle.

Social media was seen as exacerbating both division and young people’s stress about the world and the future:

You’re most likely to talk about first or read about [world issues] on social media. That’s why I think they impact youth mental health more, because it brings a sense of panic in you.

#### 2. The pressures we face: “so many people expect things from you.”

Within the context of the world young people live in, participants spoke at length about the pressures they experience including continuous pressure to be productive and build successful adult lives. Discussion centred on how this pressure impacted them, including a need to achieve, constantly plan for ‘what’s next’, and feeling daunted by pressure to map out clear career paths:

It is a lot of pressure to be honest… you got everybody just telling you, you got to do this next year, like you got your family you got your teachers, and you got like the principals and shit too.

Discussion across the workshops suggested that pressure to achieve originated largely at school and at home. Participants often felt teachers had high expectations of them to keep up with workloads and achieve excellent grades, warning them about the repercussions of failure. They felt that schools reinforced social stratifications between those who were successful and those who were not, with a sense of frustration that ‘success’ was used to define people’s worth and contributed to fear of failure.

I feel like the fear of failure is so common as well. People are just scared to try new things.

Participants also described significant pressure from parents, with many saying they felt overwhelmed by these expectations. Pressure from parents was particularly pervasive for participants from immigrant families and Pasifika young people who recognised their parents’ sacrifices and felt that they owed it to them to make them proud:

To your parents, when you don’t reach that level. It’s not just you failing with yourself, it feels like you’re letting them down as well.

Others held a more cynical view of why their parents needed them to succeed, emphasising the social status invested in their achievements.

Parental egos. Most parents I’ve noticed, especially like my dad, he’ll use me as like a token piece… It’s so much parental pressure.

Many participants also described an expectation that they be grateful for their circumstances, resulting in feelings of shame for struggling with the pressure they were experiencing. For many, particularly the children of immigrants, this expectation was connected to the sacrifices and struggles of their parents:

If your parents didn’t do well in school, if they had a rough upbringing, they’re expecting you to change that around and change the pathway for your future and then leave a good legacy for your kids.

Some participants did describe parents who were supportive, and helped protect them from pressure, however these stories were rare. The expectations from schools and parents appeared to have been internalised, with several participants outlining their own investment in success and how this contributed to their mental health challenges:

Any setback towards that goal, it’s completely damaging towards how you feel about yourself. So, you’d be like, ‘I have to get here, here’s my track to get here. I didn’t get that, I failed.’

Participants elaborated on how the pressure seemed to permeate all aspects of their lives as they forced themselves to reach the standards they felt were required. This played out in their peer interactions across social media.

It’s like this productivity culture on social media, like, you know, get up at 5am and go to the gym, do all my errands… The more you watch it, the more it gets put on your feed and the more you’re just like, ‘Oh my gosh, I’m a shit person because I don’t do this’.

Participants expressed frustration that the pressure to succeed did not allow for the constraints and barriers that prevented many young people from doing so; for example, being expected to maintain their grades through the COVID-19 pandemic while school and university classes were held online. Other participants spoke about how traumatic experiences earlier in life including childhood trauma, sexual assault, and neglect impacted them in the present, causing significant stress and impacting their capacity to meet the high standards they felt society set for them. Participants who identified as disabled spoke about experiencing significant demands with little understanding of the additional barriers they might face, or conversely being given few opportunities to show what they were capable of. Some spoke about how they were also expected to do well as school while working long hours to provide financial support to themselves and their families, while others spoke about pressure to care for younger siblings:

I’m the only person who’s working so I financially support my whole whānau and myself. Which means I’m working sometimes more than 40 h a week on top of the school and it doesn’t really give me time to catch up with schoolwork. I’m surprised that I’m like still in school to be honest.

These barriers highlighted the inequities between different groups of young people, some of whom had limited access to financial resources.

And the impact that that has on the available opportunities. You’re like, ‘I could do this’, but I can’t because there’s no funding for these opportunities.

Across most workshops, participants described a feeling of exhaustion due to this. Many wished for ‘space’ and time rather than having to constantly focus on the next thing:

I think it affects us because you know, we’re tired throughout the day and we’re obviously more prone to getting stressed out, and you know saying things we don’t want to say and doing things we don’t want to do.

As with the previous theme, participants expressed some frustration with what they saw as the lack of understanding the previous generation had for the challenges they faced in striving for a successful future, and unrealistic expectations of them, point out that older generations have simpler clearer paths to job security than today’s young people.

Importantly, a few participants acknowledged positive impacts of pressure, including seeing academic pressure as motivating and discussing how financial and familial pressure had increased their coping strategies in later adolescence and young adulthood:

I guess you can look at it as if you’re becoming more independent. When you grow up fast, you learn how to deal with things that other people don’t really know how to deal with, so that’s a positive.

#### 3. The connections we need: “how well do you know them?”

Social connection was identified by participants as a central issue affecting young people’s mental health. While participants acknowledged the value of being part of social groups and communities, they also emphasised the challenges of negotiating the complex social world they inhabited.

For many participants, friendships and peers were a common source of connection and social support, providing positive interactions including safe and supportive conversations and fun experiences. These were seen to help young people manage stress and other negative experiences.

I’d say being around friends too. ‘Cause when I come to school in the morning and I’m moody and I get to school and I just start laughing and it makes it feel better.

Participants described difficult periods, including during COVID-19 lockdowns, when they were not connected with friends and the challenges this had posed for their mental health:

The 12 to 24 period is a really important period mentally for interacting with people…I think being stuck at home during that time, and not making social bonds, the isolation is not helpful.

While friendships were recognised as an important source of support for many young people, participants conveyed difficulties negotiating and maintaining these. This included immense pressure to conform to social norms and to ‘fit in’ to particular friendship groups.

I think that can lead on to mental issues. Like you are trying to do everything you can to be a part of that group.

Conflicts and transitions in friendship groups were a major source of stress in their lives. Participants acknowledged the risks of *“toxic relationships”*, *“negative people”*, and dysfunctional friendship groups both on and offline that caused unhappiness amongst young people:

Like you feel like shit hanging out with someone and then like you get somewhere else and they’re all good.

Participants also spoke about the way that young people sometimes felt overwhelmed by their social lives, and the pressure to be around friends and having to manage the complexities of these relationships all the time:

You’re constantly socialising, making sure that you look fine, making sure that you’re not acting like a weirdo, making sure that people aren’t looking at you, or you’re not saying something dumb, or that you are keeping up appearances… it’s quite overwhelming.

The topic of conducting relationships via social media was discussed frequently in the workshops in relation to its role in affording both connection and isolation. Many participants discussed the value of online spaces to form and foster connections, through social media and gaming platforms. Many spoke to the value of these online spaces for fostering deeper connections or discussing sensitive issues:

Talking to my friends online, it’s a lot easier than talking in person…like you can send something to them and only they get it.

However, many participants acknowledged the challenges of managing online relationships and spoke about how it could be difficult to judge the authenticity or *“realness”* of a person online. They spoke about the difficulty of balancing the view that they had of people in *“real life”* with their online images and trying to work out what was “real” or what was “fake”:

You might know them, but how well do you know them? Because you get different sides of a person, the physical and you got the digital person. Those two people, those are almost two different people.

Some participants spoke about how social media left them feeling more disconnected and how online interactions were taking away from ‘real’ connections and emotions.

It’s strange sitting on a bus right next to someone and you don’t even look at them. Don’t talk to them. You’re both on your own phones doing your own thing, even though you’re not even separated by anything.

Outside of conversations about social media, many described a profound feeling of isolation and disconnection. For some participants this was linked to specific circumstances, like living rurally. Some participants spoke about broadly feeling like they moved through a world in which people did not look at each other or engage in genuine interaction:

Think it’s pretty ignorant how… we walk past like, 1000s of people a day and act like they’re not there. We don’t interact with other humans like we should.

Discussion across the workshops suggested that family relationships remained important. Participants pointed out both the potential positive impacts of family, including having close relationships with parents and feeling understood by siblings who had experienced the same things as them. However, family was also seen as a potential source of distress for young people:

I think it can either be a really positive thing for some people or a really, really not positive thing depending on the family environment you have.

Some participants elaborated on aspects of family life they found particularly challenging, including family conflict and financial strain. Many participants conveyed that it was difficult to speak openly with their families about the things that distressed them in their lives, referencing what they saw as a generational divide and a lack of understanding by older family members about issues that affected their mental health. Interestingly, outside of the family environment, many participants also gave voice to a broader inter-generational disconnection:

I feel like it’s a young people’s thing. Like beyond a certain age bracket…toughen up is the motto…You go home, and they’re like “Toughen up, what are you crying for?”

Participants from a range of immigrant backgrounds spoke about the way that mental health issues were stigmatised in their cultures, making it difficult for the older generation to accept the legitimacy of their emotional struggles:

I tried talking to my mum once about it and it just turned into an argument. It just didn’t work out. And then she called me crazy and just said it wasn’t normal.

Participants recognised the value of feeling part of communities, including sports teams, school communities (often characterised by forming relationships with supportive teachers), clubs, and shared community identities. Some conveyed a longing for a community to which they could belong.

Not having [a community] can have a negative impact. Being part of a dysfunctional one can have a negative impact. But being part of a positive community is perhaps one of the most beneficial ways to heal someone.

Importantly, regardless of the source of the connection, participants spoke about the importance of high-quality connections which were characterised by stability, listening, acceptance, and good energy:

I’m very grateful because I have people in my life who are consistent, and they check in.

#### 4. Finding our path: “you’re like trying so hard”

Participants discussed the challenges they face as they attempt to find their path within their broader social context, including ongoing challenges of forming an identity and the stressors they encounter as a result. Across all the workshops there appeared to be recognition that having a clear and positive identity helped to protect young people’s mental health:

I think identity. How confident you are in yourself, the place you belong, whether it be family, church, communities, and stuff like that.

Nonetheless, participants described how young people struggled to find and maintain a positive identity, indicating that identity formation was a complex and burdensome task requiring continuous effort. Many saw identity as *“something we’re all on a journey to doing.”*

Interestingly, participants varied in their perception of identity as a fluid or fixed state. Some participants described themselves as *“chameleons”*, adapting to the situations and *“what everyone’s doing”* around them. Others saw identity as being an ultimate state that a young person should aim to achieve and fully realise, contrasted with the *“negativity of not knowing who you are.”* These participants emphasised the importance of ‘being real’ or ‘authentic’ and ‘knowing yourself’. Some spoke of the importance of getting your identity ‘right’:

Because it’s like, what if you’re wrong? What if you’re right? They’re like, ‘Are you sure?’ Make sure that you have concrete foundation before you start saying stuff.

Many described this as a source of pressure:

Having to think about how you express yourself, and what parts of yourself you express within a particular context, is very overwhelming…. You’re presenting like a very well-orchestrated, put together version of yourself that no human on this planet is innately.

Common strategies to *“figure out who you are”* included spending time on your own and opportunities to explore and express your identity without fear of repercussion. However, they also conveyed that many young people lack freedom to truly explore their identity:

They’re limited by the rules and regulations and structures that defines who they can and cannot be. So when they’re trying to navigate things like their sexual identity, or their gender identity, or their even their cultural or religious identity, it’s done in a very confined, rigid, thought out manner.

It was also evident from discussions that participants recognised and, in some cases, connected to, a wide variety of identities available to them in Aotearoa/NZ society. Many of them spoke openly about their own identification with various groups, many of which are marginalised in relation to dominant Aotearoa/NZ society. Participants felt that Aotearoa/NZ is not always the tolerant society it might appear to be, noting that while some people had become more open there were still many sectors of society where different identities were not acceptable:

We’re in a transition space as a society. Certain groups, or the younger generation, are becoming more accepting of different expressions of identity and sexuality, gender, sexual orientation, and so I feel like…. slowly, we’re getting more acceptance for diverse groups. But there isn’t necessarily the safety and the protections and security around that.

Discussion offered numerous examples of how racism, colonisation, sexism, homophobia, and transphobia were still very much a part of many young people’s lives, presenting challenges to claiming their own identity. For some young people this included bullying, cyber-bullying, and overt threats usually described as having occurred earlier in adolescence or in childhood. Despite the protections given to Māori under the Treaty of Waitangi, Māori participants spoke about how they still struggled to have their culture recognised and the additional challenges of forming an identity as rangatahi. They spoke about the impacts that subtle forms of colonisation and racism, for example having to fend off challenges to your right to an identity, impacted on their sense of who they were:

Since I’m pretty much as a white Māori as it can get, it can lead to people assuming, “Oh, you won’t know anything about dah, dah, dah.”

Participants also captured the everyday challenges young people experienced in relation to their gender and sexual identity:

I know my partner … Because he’s transgender, he got a lot of shit from people coming up to him and being like, “So what are you? What are you?”

Importantly, despite these challenges, participants often spoke about how feeling connected to both their own identity and the communities associated with them, such as the LGBTQIA + community or their iwi, were hugely beneficial for their mental health, giving a sense of belonging and understanding of who they are.

Participants also highlighted identity threats from the social representations of young people in Aotearoa/NZ society, and frustration with the way that the adult world portrayed them. They felt that young people’s mental health was affected by the negative stereotypes of youth and the current generation:

Young people are getting a bad rap, especially the media reinforces that. The media is, like, creating this narrative that like all groups of young people hanging around together are loitering or they are doing bad things.

Discussions referenced the difficulty of getting the adults around them to respect their identities, or even to acknowledge them as people with agency:

As a young person, it kind of just always feels like you’re fighting for your power.

In this atmosphere in which young people were acutely aware of their own identity struggles, there also appeared to be an awareness of the potential to offend others by not being sensitive to their identity in a broader environment which, as participants acknowledged, had little tolerance for offence. Some participants spoke about how they felt anxious about the possibility of offending their peers, while others spoke of judging others for their lack of sensitivity around these issues:

There are some people that we’ve cancelled and then we all just stop liking them because they said something or did something really bad.

The struggles associated with identity were seen to be exacerbated by social media. There were some conversations about bullying online, however participants also discussed more subtle challenges to managing their identity online. They described what it was like to curate an online identity:

It’s almost this brand image that you create for yourself. But then like, you don’t really have much expression of your identity beyond that. I become almost like a caricature, and it’s like a narrative that you create online over the time, and then that becomes you.

They expressed how this contributed to confusion about who they were and sometimes a sense of alienation from their own sense of self:

Building a second personality is like a downfall. Because then you don’t recognize yourself as like, “Am I really the person I am online?” Or is it like, “Am I really the person that I am in person?”

Many participants highlighted how their identity interacted with social expectations. These included expectations of how you ‘should’ behave and look, particularly in connection to body image. Participants of all genders had a strong sense of how their body and physical appearance was being interpreted by others, often exacerbated by comparison to others online. Young women often discussed feeling sexualised, with some noting this could become internalised as they *“present themselves in a certain way to please the male gaze.”* As one participant said:

I normalized my own discontent towards my body. I just wished that I was white enough and I was skinny enough.

## Discussion

In this study, we explored what young people from Aotearoa, New Zealand believe impacts their mental health both positively and negatively, highlighting young people’s understanding of the social determinants of mental health and their attempts to deal with these. 176 young people participated in one of 19 workshops, which were transcribed and analysed qualitatively. Four themes were identified, which centred on (1) challenges they perceived in the world, (2) the pressure of expectations, (3) finding social connection and (4) developing and protecting their own identities. This analysis identified that participants in this research thought broadly and systemically about how mental health challenges develop and the core issues at play.

Several of the issues young people reported here shared broad similarities to those described in past research. Young people’s perceptions of the future as bleak, particularly in connection to existential threats, has been increasingly identified as a factor that influences their mental health and wellbeing [49]. While anxiety about the future has long been identified as playing a role in mental health [50], recent evidence consistently identifies links between threats like COVID and war with young people’s anxiety about the future (e.g., 51, 52). Globally, young people have described mental health challenges associated with COVID [53–55] and climate change [56, 57]. Interestingly, participants in this study identified that the state of the world, including witnessing inequality and polarization, impacts their mental health beyond increasing anxiety about the future. Many expressed feelings of being overwhelmed and fatigued by their current experiences, including difficulties making ends meet, and frustration and anger at inequality. Stress and pressure have been consistently identified as key issues impacting youth wellbeing (e.g., 58), and it is perhaps unsurprising in the current economic state [59] that financial pressure has emerged so strongly as an issue of core importance to Aotearoa/NZ’s youth. International evidence has also identified that job insecurity and financial status are associated with youth mental health [60]. Perception of support and connection have been consistently linked to mental health around the world (e.g.,^61^, ^62^, ^63^), particularly for LGBTQIA + young people (e.g., 64) and, in Aotearoa/NZ, young Māori and Pasifika people (e.g.,^65^). Young people in our study indicated nuanced understandings of how they are impacted by navigating an increasingly complex social world, in which many social connections are fostered and facilitated online. Consistent with past research [66], social connections online were characterized as having the potential to increase a sense of support and reduce feelings of isolation.

Participants discussed nuanced relationships between identity and mental health. Identity formation has been a focus of youth developmental literature for several decades, beginning with the work of Erik Erikson [67], and continuing into an evolving understanding of identity development, acknowledging both the prolonged duration and increased complexity of contemporary identity formation [68–71]. As such, it is developmentally appropriate that topics related to identity and how to navigate mental health challenges with agency were common in our workshops. Interestingly, however, participants in the present study often focused on the notion of knowing yourself as a finite state, and the importance of being ‘right’ about your identity. It is possible that constraints in young people’s worlds, including social pressure to ‘fit in’, the curated personas they are expected to portray on and offline, and limited social acceptability of certain identities produce constraints on true exploration which may limit young people’s capacity to deeply and holistically explore and form their identity. Future research on the process of identity formation for today’s young people, and how social discourses around authenticity and identity impact identity exploration, will be valuable for understanding how youth navigate identity and its impacts on their mental health.

Much of recent literature on youth mental health, particularly that which has been reported in the media, has centred around the impact of social media [32]. In some ways a focus on social media is intuitive, as the introduction of social media has aligned with rising rates of mental health challenges [72]. However, across discussions in this study, social media clearly emerged as an amplifier of other influences that impact mental health positively and negatively both on and offline, rather than as an independent entity affecting mental health. For example, through increasing exposure to information about the world social media increased young people’s sense that the world is unsafe and the future is uncertain. For others, social media may provide an opportunity for advocacy and reduce a feeling of hopelessness. For many, social media promoted comparison, unattainable lifestyles, and increased the stress and pressure young people feel in their day to day lives. Through impacts on how young people connect, social media can increase both a sense of support and a sense of isolation. Social media can amplify challenges of forming and exploring identity and can also provide spaces where young people can safely explore identity and find a sense of belonging.

Young people are diverse in their perspectives, and there is danger in characterising a singular ‘youth perspective’. Notably, each of these themes were identified in discussions with young people of diverse socio-economic status, educational background, gender, sexuality, and ethnicity. Specific issues tended to emerge more strongly in some discussions than others, and examples offered also tended to differ – for example, while even higher income young people expressed concerns about economic futures, lower income students spoke more about how their current financial situations impacted current mental health and wellbeing.

### Implications

Present findings highlight that, for many young people in Aotearoa/NZ, the challenges they face feel overwhelming and insurmountable. For those working with young people, including in mental health care or in other settings such as education, it can be challenging to navigate preparing young people for the world they face, addressing mental health challenges that impact quality of life or functioning, and honouring the very real challenges many young people experience. In this study, young people highlight a feeling that ‘older’ generations do not understand their experiences or take them seriously, which they connected to feeling helpless about the possibility of change. It is important to highlight for practitioners and all people that interact with young people that feeling understood [73] and validated [74] have positive impacts on wellbeing, including for marginalized youth [75]. Listening deeply to young people’s concerns, taking these seriously, and being open to their experiences are likely to be very helpful interventions – possibly even therapeutic in themselves, if an older practitioner can bridge the ‘inter-generational divide’ many participants describe. Acceptance of young people’s identity exploration, an essential stage of positive human development [76], is another role those working with young people can play – countering expectations many young people feel that adults have negative perspectives on them and other young people. It is clear, also, that many of the concerns impacting young people’s mental health at a public health level lie outside of the traditional scope. Mental health practitioners and all professionals who work with youth must consider how our professional knowledge can be appropriately applied to addressing many of these broader concerns including social determinants [77], as well as how we can acknowledge these existential threats within the scope of our roles.

### Limitations

In this study, we sought to explore young people from Aotearoa New Zealand’s perspectives on what influences youth mental health. While our workshop design allows for rich conversations, the transcription process prevents us from consistently identifying the individual voices of specific workshop participants and assigning pseudonyms or participant numbers. Notably, while we worked with a representative sample of young people in this study, we only conducted workshops in the Auckland and Northland regions of Aotearoa/NZ and there may be regional variances outside of these areas. It is important to highlight that young people are not homogeneous and represent a range of experiences, contexts, backgrounds, and beliefs which must be embraced to fully understand the nuance of their experiences.

## Conclusions

The findings of this research demonstrate young people’s awareness of several systemic influences which play a role in their wellbeing. Importantly, these findings also identify key future directions for both research and our response to young people’s mental health. This study highlights the importance of targeting the issues that matter to young people and supporting them in their attempts to manage their mental health through challenging the social status quo, resisting pressure, findings points of connection and protecting their identities.

Future research exploring whether and how these themes differ across global contexts can help explore whether declining mental health for young people around the world are related to shared and/or unique factors including the themes described by young people in Aotearoa/NZ. Research should also focus on identifying prevention and intervention approaches that clearly target these priority areas.

## Electronic supplementary material

Below is the link to the electronic supplementary material.

Supplementary Material 1

## Data Availability

The datasets generated and/or analysed during the current study are not publicly available to protect the confidentiality of our participants given the sensitive nature of this research, but may be available from the corresponding author on reasonable request.
