# Supplementary material for: “We’re living in a world that wasn’t built for us”: A qualitative exploration of young New Zealander’s perspectives on socio-ecological determinants of declining youth mental health
Source: BMC Public Health. 2025 May 5;25:1648. doi: 10.1186/s12889-025-22618-2 (PMC12051270; doi:10.1186/s12889-025-22618-2)
Supplement: Supplementary file 1 — Supplementary Material 1 [file 12889_2025_22618_MOESM1_ESM.docx]

**Appendix 1: Surveys**

**Self-Reflection 1**

*(for individual completion at opening of workshop)*

Age:

Gender:

Ethnicity:

Do you identify as a member of the LGBTQIA+ / Rainbow community? YES NO

How do you identify your sexuality?

Are you currently attending school or University? YES NO

If yes, what year are you in?

Is it part or full time?

Are you currently working? YES NO

If yes, please indicate how many hours:

Are you currently volunteering? YES NO

If yes, please indicate how many hours:

Do you have any personal experience with mental health challenges? YES NO

If yes, please indicate if:

*Own experience Family member with experience Friend/peer with experience*

What does mental health and wellbeing mean to you?

From a scale of 1-10 (1 = Awful, 10 = Excellent), how do you think young people’s mental health, on average, is doing at the moment in Aotearoa, New Zealand?

Please explain why you chose that number.

What things are good for young people’s mental health?

What things are bad for young people’s mental health?

What could make young people’s mental health better?

Do you have any other thoughts or ideas related to these questions?

**Self-Reflection 2**

*(For individual completion at end of workshop)*

Is there anything else you would like to say?

Was there anything else you wanted to share?

Do you have any feedback for us about this workshop?
